# Latent class analysis of the social determinants of health-seeking behaviour for delivery among pregnant women in Malawi

**DOI:** 10.1136/bmjgh-2018-000930

**Published:** 2019-03-30

**Authors:** Rachel R Yorlets, Katherine R Iverson, Hannah H Leslie, Anna Davies Gage, Sanam Roder-DeWan, Humphreys Nsona, Mark G Shrime

**Affiliations:** 1 Department of Plastic & Oral Surgery, Harvard Medical School, Boston Children’s Hospital, Boston, Massachusetts, USA; 2 Program in Global Surgery and Social Change, Harvard Medical School, Boston, Massachusetts, USA; 3 Department of Surgery, University of California Davis Medical Center, Sacramento, California, USA; 4 Department of Global Health and Population, Harvard TH Chan School of Public Health, Boston, Massachusetts, USA; 5 Integrated Management of Childhood Illnesses (IMCI), Ministry of Health, Lilongwe, Malawi; 6 Center for Global Surgery Evaluation, Massachusetts Eye and Ear Infirmary, Harvard Medical School, Boston, Massachusetts, USA

**Keywords:** health systems, health services research, maternal health, public health, latent class analysis

## Abstract

**Introduction:**

In the era of Sustainable Development Goals, reducing maternal and neonatal mortality is a priority. With one of the highest maternal mortality ratios in the world, Malawi has a significant opportunity for improvement. One effort to improve maternal outcomes involves increasing access to high-quality health facilities for delivery. This study aimed to determine the role that quality plays in women’s choice of delivery facility.

**Methods:**

A revealed-preference latent class analysis was performed with data from 6625 facility births among women in Malawi from 2013 to 2014. Responses were weighted for national representativeness, and model structure and class number were selected using the Bayesian information criterion.

**Results:**

Two classes of preferences exist for pregnant women in Malawi. Most of the population 65.85% (95% CI 65.847% to 65.853%) prefer closer facilities that do not charge fees. The remaining third (34.15%, 95% CI 34.147% to 34.153%) prefers central hospitals, facilities with higher basic obstetric readiness scores and locations further from home. Women in this class are more likely to be older, literate, educated and wealthier than the majority of women.

**Conclusion:**

For only one-third of pregnant Malawian women, structural quality of care, as measured by basic obstetric readiness score, factored into their choice of facility for delivery. Most women instead prioritise closer care and care without fees. Interventions designed to increase access to high-quality care in Malawi will need to take education, distance, fees and facility type into account, as structural quality alone is not predictive of facility type selection in this population.

**Key questions**

**What is already known?**

- In Malawi, where over 90% of births are facility-based and high-quality delivery facilities exist, progress towards Sustainable Development Goals 3 for reducing maternal and neonatal mortality relies on mothers using high-quality delivery services, but little is known about why women choose to deliver in a particular health facility and how quality affects that decision.

**What are the new findings?**

- Our revealed-preference latent class analysis conducted with a large, nationally-representative, geospatially-linked dataset of women who gave birth in Malawi from 2013 to 2014 provides key insights into what factors are predictive of facility selection for delivery.
- Our analysis shows that, in a setting where maternal and neonatal mortality is high, the majority of women are using services that are close and free and that the quality of the services, as measured by a structural capacity metric, is not predictive of their care utilisation.

**What do the new findings imply?**

- The knowledge that high-quality delivery services can reduce maternal and neonatal mortality combined with this new evidence that most Malawian women are not using high-quality services can inform effective policy intervention.
- In order to connect women with high-quality care to reduce mortality ratios, mechanisms are needed to either facilitate free transportation to high-quality facilities that are further away or to increase the demand for higher quality care at local, more accessible facilities.

## Introduction

Despite reductions in maternal and neonatal mortality over the past 15 years, more progress is needed to reach the Sustainable Development Goals (SDGs), particularly in sub-Saharan Africa.^1^ The SDGs call for a reduction of the maternal mortality ratio (MMR) to fewer than 70 deaths per 100 000 births and of neonatal mortality ratio (NMR) to fewer than 12 per 1000 live births by 2030.^2^ These goals present a strong opportunity for Malawi, a country with an MMR of 439 maternal deaths per 100 000 live births and NMR of 27 neonatal deaths per 1000 live births.^3^

Historically, efforts to improve these outcomes have focused on increasing facility-based deliveries, but evidence suggests that increased access to care may not reduce mortality.^4^ Malawi has a high prevalence of facility-based delivery, with only about 9% of women delivering at home (ranging from 5% of urban women to 12% of rural women),^5 6^ which may be partly attributed to national policy that prohibits traditional birth attendants (TBAs) from practising. Despite the subsequent increase in utilisation of the formal sector for deliveries after the TBA ban in 2007, overall neonatal mortality has not changed concurrently: only women with access to a high-quality health facility saw a reduction in newborn deaths, whereas women with access to low-quality health facilities saw no improvement.^7^ Furthermore, other studies have shown an association with poor quality of care in facilities and a higher risk of neonatal mortality in Malawi since the implementation of this policy.^8^

This existing evidence illustrates what is becoming increasingly apparent globally: both access and high-quality services are required to improve outcomes.^9^ Furthermore, this focus on quality of facility-based care must be merged with an understanding of how women choose where to deliver. Previous studies have found that facility-level factors, such as location and cost, are significant predictors of facility-based deliveries in low- and middle-income countries.^10–13^ Women’s sociodemographic characteristics, such as wealth, education and urbanicity, have been positively correlated with facility-based births in several East and Central African countries, including Malawi.^13–15^ However, less is known about how a woman chooses a specific facility for delivery and how quality factors into this decision.

Perceived quality has been shown to factor into a woman’s decision of whether to deliver at any facility,^10–12 16^ but less is known about the role of measured quality on a woman’s specific facility selection. Previous stated-preference studies using discrete choice experiments have shown women in Ethiopia and Tanzania value technical attributes associated with quality, such as the availability of medicine, equipment and skilled providers, when choosing a delivery facility.^12 16 17^ Furthermore, a preference for quality of care may be associated with individual characteristics: Larson *et al* 12 found an association with higher education and higher wealth with women’s preference for certain markers of quality, such as provider medical knowledge, in Tanzania. However, there is less evidence to support this from revealed-preference studies. One revealed-preference study in Ghana showed no association between use of the nearest facility and its measured quality of care, aside from when that nearest facility was providing below the standard of care for emergency obstetric services.^13^

This study aimed to determine what factors contribute to a Malawian woman’s choice of delivery facility, with the goal of informing effective policies to improve maternal and neonatal mortality. In the context of The *Lancet Global Health* Commission on High Quality Health Systems in the SDG Era, we hypothesised that quality of the health facility, as measured by its readiness to deliver basic obstetric care, would be a predictor of chosen facility type for delivery. As individual attributes, such as demographic characteristics, have been found to be predictive for facility preference in previous studies, we also predicted heterogeneity within the population.

## Methods

### Study sample: women’s deliveries

Primary data about individual women and their deliveries were obtained from the 2013–2014 Millennium Development Goal Endline Survey (MES),^18 19^ a nationally-representative household survey that used a multistage stratified sampling strategy to include households within enumeration areas (EAs) identified by the 2008 census. Locations of EAs in the MES were obtained from the Malawi National Statistical Office, 2008 Malawi Population and Housing Census, 2013 update.^18^ Responses were weighted for national representativeness. More detail on the MES survey has been published elsewhere.^8^

**Figure 1:**
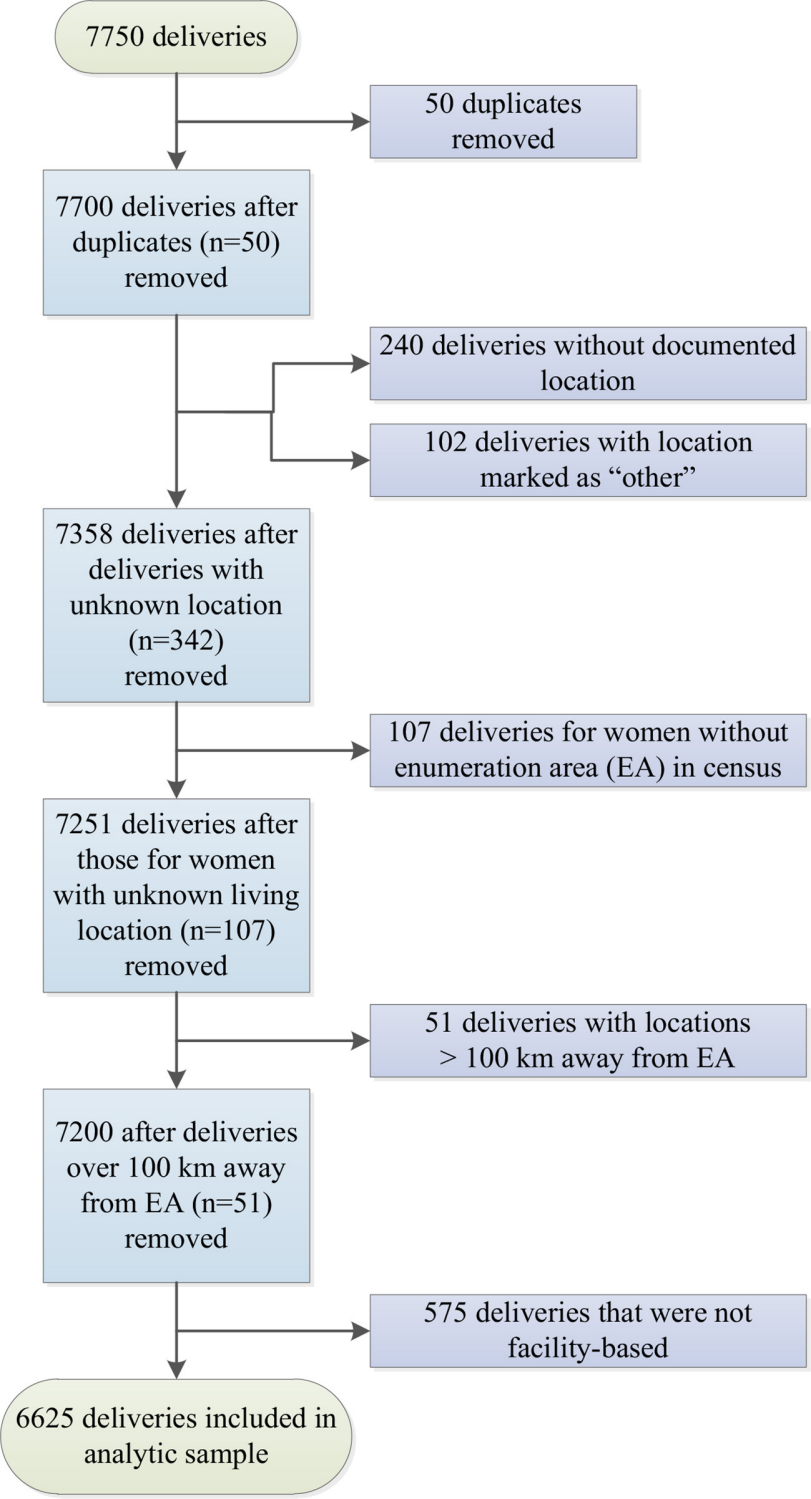
Application of exclusion criteria to create analytic sample (n=6625).

A total of 7750 deliveries was captured by the MES; women were surveyed about their most recent pregnancy (if more than one) in the past 2 years (2013 and 2014). Exclusion criteria for this study included the first entry of any duplicated record (n=50), women with no documented delivery location (n=240) or a reported delivery location that could not be matched to the Malawi Service Provision Assessment (SPA)^19^ facility types (eg, *‘*Other’; see next section on delivery facilities) (n=102), an EA with a location that could not be matched to the census (n=107) and delivery more than 100 km away (n=51) (figure 1). Because of the legal barriers to delivering with a TBA and because fewer than 10% of women in the survey reported delivering at home, home delivery was also excluded (n=575), leaving an analytic sample of 6625 women.

### Delivery facilities

Health facility data, including geographic location, were obtained from the 2013 Malawi SPA,^19^ a census of the health system^20^ that includes a detailed audit of facility resources and clinical practices, including whether fees are charged for labour and delivery services.

The MES asks women about the type of facility where they delivered (eg, government health centre, mission hospital and private maternity home), but not the name of the specific facility where they delivered. The SPA includes information on facility tier (eg, central hospital, district hospital and clinic) and management type (eg, government and Christian Health Association of Malawi). We aligned facility type responses between the MES and SPA surveys. We identified up to eight facilities of each facility type located near the woman’s EA centroid using Euclidean distance and calculated road distance to each of these in order to select the nearest facility by road. If road distance could not be calculated, we selected the closest facility of the appropriate type using Euclidean distance. Women were assigned to the closest facility matching the facility type they reported on the survey.

A choice set of six facilities was created for each woman in order to analyse her facility preferences. This included the five nearest facilities to a woman’s EA in linear distance that were providing delivery services. The sixth choice included the facility the woman matched to if it was outside the five closest facilities in the choice set. A description of the choice set and associated characteristics can be found in table 1.

**Table 1:** Description of facilities in choice set

Facility choice	Definition	Deliveries at each facility choice		Distance from EA to facility		Basic obstetric readiness score	
		n	%	Median	IQR	Median	IQR
Rural women							
1	Closest facility with delivery services to woman’s EA	3144	54.1	6.30	(3.44–9.41)	0.60	(0.46–0.71)
2	Second-closest facility	1032	17.8	12.39	(9.11–16.49)	0.60	(0.46–0.71)
3	Third-closest facility	481	8.3	16.87	(12.66–22.03)	0.60	(0.46–0.72)
4	Fourth-closest facility	273	4.7	20.61	(15.71–25.92)	0.61	(0.46–0.71)
5	Fifth-closest facility	197	3.4	23.43	(18.64–30.39)	0.58	(0.46–0.71)
	**Total**	**5127**	**88.2**	**15.98**	**(10.17–22.83)**	**0.60**	**(0.46–0.71)**
6	Facility matched for delivery, if outside of five nearest	684	11.8	35.16	(26.83–47.89)	0.74	(0.63–0.83)
Urban women							
1	Closest facility with delivery services to woman’s EA	500	61.4	1.95	(1.23–2.99)	0.74	(0.61–0.80)
2	Second-closest facility	121	14.9	5.59	(3.62–12.09)	0.66	(0.47–0.78)
3	Third-closest facility	68	8.4	10.74	(5.93–17.46)	0.60	(0.46–0.71)
4	Fourth-closest facility	36	4.4	14.25	(7.52–19.88)	0.58	(0.46–0.69)
5	Fifth-closest facility	15	1.8	17.08	(9.69–23.98)	0.63	(0.48–0.77)
	**Total**	**740**	**90.9**	**8.13**	**(3.73–17.34)**	**0.64**	**(0.48–0.77)**
6	Facility matched for delivery, if outside of five nearest	74	9.1	9.97	(8.45–18.57)	0.91	(0.69–0.91)

### Measure of facility quality: basic obstetric care service specific readiness score

The service readiness score for basic obstetric care was used as the marker for structural quality of delivery services in each health facility. The score is based on the recommended essential items needed to provide quality facility-based delivery services from the WHO Service Availability and Readiness Assessment Manual.^21^ The tracer items that compose the score include the availability of management guidelines, staff up-to-date with training and essential equipment, medicines and commodities for delivery care. The basic obstetric care service readiness score for each health facility was derived from the 2013–2014 SPA data.^19^

### Analysis

Data from the SPA and MES in Malawi were used to directly link characteristics of facilities to the delivery choices made by a nationally-representative sample of women who gave birth in 2013 or 2014. We hypothesised that facility characteristics could predict choice^22^ but that different preferences for these characteristics might exist across this cohort of women. To identify this unobserved, or latent, heterogeneity within this population, we chose to conduct a latent class analysis. Latent class analysis assumes a discrete number of segments (or ‘classes’) in the population, each with its own preference structures.^23 24^ In the context of this study, this analysis allows us to identify the different utilities for facility characteristics (as revealed in women’s facility type selection), determine the number of latent classes, calculate the probability of each woman belonging to each group or class and, finally, summarise the sociodemographic characteristics of the women likely to belong in each class. Following random utility theory, we assign the utility for woman *i* choosing alternative *f* to be^24^:

$$U_{if}=\beta_{i}x_{f}+\varepsilon_{if}$$

where $\beta_{i}$ is the vector of preference coefficients for a woman for each facility-level characteristic, and *x* is the vector of facility-level characteristics (eg, obstetric readiness and fees). The error term, $\varepsilon_{if}$, is assumed to follow a Gumbel Type 1 distribution. With this assumption, the probability of a woman choosing an individual facility is:

$$p_{i}(facility\;choice=1)=\frac{e^{\beta_{i}x_{1}}}{\sum_{f=1}^{F}e^{\beta_{i}x_{f}}}$$

where *F* is the total number of facilities in a woman’s choice set. The value of each β in the $\beta_{i}$ vector is identical for each woman within a class but can take different values across classes. The probability of each woman belonging to a class is:

$$p_{i}(class=1)=\frac{e^{\gamma_{1}\delta_{i}}}{\sum_{q=1}^{Q}e^{\gamma_{q}\delta_{i}}}$$

where γ is the vector of logistic regression coefficients on sociodemographic variables, δ is the vector of examined sociodemographic variables and *Q* is the total number of classes in the latent class analysis.^23^

The variables included in the analysis are defined in the data dictionary online supplementary appendix table 1. Four facility-specific variables were selected *a priori* based on literature suggesting that accessibility, quality and out-of-pocket payment factors into facility selection, as cited above. Twenty-four individual-specific variables were chosen based on prior literature and author consensus and were tested stepwise. One hundred and ninety-four combinations of individual-specific variables were tested, with the Bayesian information criterion (BIC) informing the selection of the best-fitting formula. Sensitivity analyses were performed. After the model was selected, it was tested with 2–6 latent classes to determine the likely number of underlying preference structures; BIC informed.

10.1136/bmjgh-2018-000930.supp1 Supplementary data

It should be noted that this is a revealed-preference latent class analysis: women reported characteristics of their deliveries retrospectively. Therefore, this is an analysis of the facility types that women chose, which reveal preferences, but this is not assumed to be the same as each woman’s stated-preference.

Entropy, an indicator of quality of the model, was calculated to determine the separateness of the classes:

$$E=1+\frac{1}{N\;ln(Q)}\left(\sum_{i=1}^{N}\sum_{q=1}^{Q}(P(R_{i}=q|W_{i})ln(P(R_{i}=q|W_{i})\right)$$

where *Q* is the number of classes, *N* is the sample size, *R* is the latent class indicator variable for each woman *i*, $W_{i}$ is the vector of latent class indicator variables for each woman *i* and the probability Extra close brace or missing open brace is generated from the final model.^24^

Lastly, to evaluate the potential bias introduced by random effects at the facility level, given that data are clustered by facility, a multilevel model was examined.^25^

The analytic dataset was created using Stata V.14.1. Geographic distances were calculated based on Google Maps using Python 3.6.1. Statistical analyses were performed using R V.3.4.0 (R Foundation for Statistical Computing, Vienna, Austria) gmnl package.^15 26^

## Results

Of the 6625 unique respondents included in the analytic sample, most were from a rural area, Christian, married and literate. The three lowest wealth quintiles were slightly over-represented in our sample. The mean age for women was 26±6.56 years and, for women who reported having a partner (84.2%), mean partner age was 32±8.08 years. Most women (75.5%) were multiparous. Less than half (43.6%) reported this pregnancy as unintended. Almost all women (99.5%) attended at least one antenatal care visit, and nearly half (45.6%) attended four or more visits. Most pregnancies (78.7%) had 0 or 1 risk factor, and caesarean delivery was predetermined for only a small proportion (1.6%) (table 2).

**Table 2:** Respondents’ demographic characteristics (n=6625)

Characteristic	Mean	SD
Age		
Woman’s age at delivery	26	6.56
Partner age	32	8.08

	n	%
Urban	814	12.3
Ethnicity		
Chewa	1890	28.6
Lomwe	1167	17.7
Yao	914	13.8
Ngoni	826	12.5
Tumbuka	670	10.1
Sena	350	5.3
Tonga	208	3.1
Nkhonde	109	1.6
Other	474	7.2
Religion		
Christian, other denomination	2846	43.0
Catholic	1133	17.1
Muslim	943	14.2
Church of Central Africa Presbyterian	875	13.2
Seventh Day Adventist	377	5.7
No religion	236	3.6
Anglican	180	2.7
Other religion	35	0.5
Education		
None	680	10.3
Primary	4669	70.5
Secondary	1198	18.1
Higher	77	1.2
Literacy		
Can read	4444	67.1
Cannot read	2158	32.7
Blind or visually impaired	3	0.05
Missing	20	0.003
Wealth		
Poorest	1514	22.9
Poor	1473	22.2
Middle	1407	21.2
Rich	1164	17.6
Richest	1067	16.1
Marital status		
Not currently married	1048	15.8
Pregnancy		
Primiparous	1620	24.5
Multiple	151	2.3
Unintended	2886	43.6
Mistimed	2231	33.7
Unwanted	655	9.9
Antenatal care		
Attended ≥1 antenatal care visit	6591	99.5
Attended ≥4 antenatal care visits	3022	45.6
Delivery risk		
0 risk factors	2152	32.5
1 risk factor	3062	46.2
2 risk factors	929	14.0
3 risk factors	465	7.0
4 risk factors	17	0.3
5 risk factors	0	0.0
Caesarean delivery predetermined	105	1.6

Note that delivery risk was calculated from five MES items for woman’s last pregnancy. Number of risk factors that each woman reported. Risk factors included maternal age <19 years, incomplete antenatal care (<4 visits), primiparity, giving birth to multiples (eg, twins) and giving birth to a very small neonate (<2500 g, or a neonate described as very small if no birth weight data).

MES, Millennium Development Goal Endline Survey.

There were 531 unique facilities included in the facility choice sets. Most women (87.9%) delivered at a public facility, 10.3% delivered at mission hospitals and 1.9% delivered at private facilities. Most women delivered at a health centre (59.7%), which constituted 77.8% of the health facilities. Only 26.2% of the total facilities charged fees for delivery services (table 3). Fifty-five per cent of women were assigned to their nearest facility for delivery, whereas 17.4% of women were matched to the next closest facility for delivery, followed by 8.3% for third closest, 4.7% for fourth closest and 3.2% for fifth closest. Seven hundred and fifty-eight (11.4%) women matched outside of her five closest options. Seventy-four per cent of women in urban EAs and 60% of women in rural EAs had a facility with a high basic obstetric readiness score (defined as greater than 0.75) within her five closest options (table 1).

**Table 3:** Characteristics of health facilities (n=531) included in the choice set

Facility type	Facilities of each type		Deliveries at each type		Basic obstetric care score		Facilities with fees	
	n	%	n	%	Median	IQR	n	%
Central hospital	4	0.8	177	2.7	0.86	(0.78–0.94)	0	0.0
District hospital	24	4.5	1604	24.2	0.76	(0.67–0.81)	1	4.2
Rural/community hospital	41	7.7	567	8.6	0.72	(0.61–0.80)	15	36.6
Other hospital	28	5.3	258	3.9	0.68	(0.65–0.80)	22	78.6
Clinic	17	3.2	38	0.6	0.55	(0.46–0.66)	13	76.5
Health centre	413	77.8	3952	59.7	0.55	(0.43–0.66)	86	20.8
Maternity	4	0.8	29	0.4	0.45	(0.33–0.58)	2	50.0
	531		6625				139	26.2

Note that ‘Other hospital’ consists of private hospitals, Christian Health Association of Malawi or mission hospitals and some government hospitals.

Based on the BIC, the latent class analysis revealed two groups of preferences for delivery facilities (online supplementary appendix figure 1). The facility-level variables retained in the analysis, which are predictive of the preferences in each class, included the facility type (SPA type), distance to the facility from the centre of the woman’s EA, the facility’s basic obstetric readiness score and whether the facility charged fees (table 4). Of the individual-level variables retained in the analysis (table 5), those most predictive of membership within a preference class were the woman’s wealth, age, education, literacy, pregnancy characteristics of being pregnant with multiples, whether the pregnancy was unwanted (a subset of unintended pregnancies) and whether a caesarean delivery was planned before labour onset.

**Table 4:** Facility-level preferences within each class

Alternative	Coefficient	Robust SE	*p-*value for difference
**Class 1**			
Facility type			
Central hospital	Reference		
District hospital	15.045	145.070	0.917
Community hospital	7.612	145.070	0.958
Other hospital type	15.416	145.070	0.915
Clinic	6.903	145.070	0.962
Health centre	15.597	145.070	0.914
Maternity	14.332	145.070	0.921
Distance to facility (km)	−6.053	0.369	<0.001
Basic obstetric readiness (scale 0–1)	−0.436	0.273	0.110
Fees (reference=no fees)	−5.737	0.565	<0.001
**Class 2**			
Facility type			
Central hospital	Reference		
District hospital	−1.053	0.267	<0.001
Community hospital	−2.243	0.259	<0.001
Other hospital type	−2.391	0.248	<0.001
Clinic	−2.860	0.309	<0.001
Health centre	−4.624	0.283	<0.001
Maternity	−22.168	4387.700	0.996
Distance to facility (km)	0.835	0.069	<0.001
Basic obstetric readiness (scale 0–1)	1.451	0/294	<0.001
Fees (reference=no fees)	−0.105	0.108	0.327

The coefficient denotes the log OR in comparison with the reference within each category and the other class. Note that ‘maternity’ facilities are few (n=4).

**Table 5:** Individual-level characteristics of class 2 compared with class 1

Alternative	Coefficient	Robust SE	*p*-value for difference
Wealth			
Poorest	Reference		
Poor	−0.072	0.102	0.482
Middle	−0.002	0.104	0.984
Rich	0.099	0.109	0.366
Richest	0.316	0.133	0.018
Urban/rural			
Rural	Reference		
Urban	−0.007	0.127	0.958
Woman’s age	0.140	0.067	<0.05
Spouse or partner age	−0.092	0.063	0.147
Education			
Primary education or below (includes preschool)	Reference		
Secondary education or above	0.485	0.096	<0.001
Literacy			
Literate	Reference		
Illiterate	−0.190	0.079	0.017
Blind or visually impaired	11.377	96.637	0.906
Primiparous	0.305	0.176	0.084
Multiple birth (eg, twins)	1.009	0.249	<0.001
Woman’s pregnancy unwanted	0.354	0.249	<0.001
At least four antenatal care visits during pregnancy	−0.008	0.131	0.951
Delivery risk score (scale 0–5)	−0.031	0.111	0.782
Caesarean delivery planned before labour onset	2.428	0.411	<0.001

Twelve individual-level variables were included based on best fit of the formula to the dataset. Note that few women (n=3) reported being ‘blind or visually impaired’. The coefficient denotes the log OR in comparison with the reference within each category and the other class.

The first class describes 65.85% (95% CI 65.847% to 65.853%) of the population. For this preference group, distance to the facility from the woman’s EA (p<0.001) and the absence of fees (p<0.001) were statistically significantly associated with the type of facility the woman selected; women in this class selected closer facilities and facilities that did not charge fees (table 4). For this class, the marginal rate of substitution (the rate at which a consumer can exchange an amount of one good for another while maintaining utility) between these factors indicates removing fees would be approximately equivalent to women moving 1.1 km closer to a facility. The second class showed a statistically significant preference for facilities with a higher basic obstetric readiness score (p<0.001), facilities that are located further away (p<0.001) and central hospitals over all other facility types (p<0.001) except for maternity facilities.

The second preference class made up 34.15%(95% CI 34.147 to 34.153%) of the population. Membership in this class is predicted by being in the highest wealth quintile—richest (p=0.018)—being older (p<0.05), having completed secondary education or above (p<0.001), being literate (p<0.05), pregnant with multiples (p<0.001), having an unwanted pregnancy (p<0.01) and having a caesarean delivery planned before the onset of labour (p<0.001) (table 5). Assuming a reference group of women with no education or primary education only, women with secondary education or higher have 1.32 times the odds of belonging to class two over class one.

Sensitivity analyses were performed to test for interaction between education level and literacy; substituting a combined variable (eg, individuals who have no education and cannot read and so on) for the individual variables resulted in an unstable model. The model was stable when the combined variable was included, but urbanicity or wealth was excluded, and when both urbanicity and wealth were excluded. Given existing literature, urbanicity and wealth were selected over the combined variable for education and literacy for the final model.

Entropy was calculated to evaluate the separateness of the classes; the value of 0.11 indicates limited separation between the first and second preference class. To examine intraclass correlation, we constructed a multilevel model. This model was unstable, but its coefficients are reported in the online supplementary appendix table 2.

## Discussion

For most women who gave birth in Malawi from 2013 to 2014, distance and fees drove their revealed-preference of facility. For these women, quality, as indicated by a measure of structural capacity, was not predictive of their facility type selection. This measure of quality was predictive of choice for only about one-third of women; these women were also more likely to be older, in the highest wealth quintile, have a secondary education or higher, be literate, pregnant with multiples, have an unwanted pregnancy and have a planned caesarean delivery before labour onset. These women were also more likely to travel further for care.

In order to reach SDGs 3.1 and 3.2 to reduce maternal and neonatal mortality,^2^ efforts have been made to identify interventions to improve obstetric care. One study found that delivery in a high-quality facility in Malawi was associated with an estimated 23 fewer neonatal deaths per 1000 live births than delivery in lower quality facilities.^8^ Malawi’s ban on TBAs^27^ encourages women to seek formal health facilities for delivery. From 2007 to 2016, there was a 15% decrease in TBAs and an 11% increase in facility utilisation. However, this did not correspond to reduced neonatal mortality, which suggests a need to raise quality, rather than to only expand access.^7^ Indeed, Malawi’s Every Newborn Action Plan prioritises an investment in improving quality of obstetric care to reduce neonatal mortality.^28^

However, availability of higher quality services cannot impact neonatal mortality if women do not give birth in high-quality facilities. Similar to our study, surveys of Malawian mothers of deceased newborns reported distance, limited or no transportation and financial burden as the factors that most commonly affect care-seeking.^29^ Most women in our study were from rural areas; 54% of rural Malawians are within 5 km of a health facility,^29^ but only 1.4% of all Malawians have a car.^30^ Malawian women have reported barriers to accessing care, or preferring closer care, because they cannot afford public transportation or the cost of fuel for an ambulance^31^; furthermore, the time to reach these far-away facilities may result in delayed or denied care if they arrive after hours.^31^ Additionally, women report a preference for nearby hospitals because the women are very busy,^32^ prefer to have relatives close-by^32^ or do not have help from their partners.^2 29 33^

Our study showed that the one-third of women whose revealed-preference was for higher quality care was more likely to be older, wealthy, literate and have a secondary education level or higher. This difference may translate to differences in neonatal mortality outcomes: most deceased neonates in Malawi were from poor homes, characterised by limited access to clean water or electricity.^29^ Additionally, most mothers of these deceased newborns were illiterate and had three or fewer years of education.^29^ Low educational attainment has also been shown to be a barrier to utilisation of antenatal, delivery or postnatal care.^32^

The finding that most women deliver in closer facilities that do not charge fees indicates the need to either bring higher quality care closer to women or to reduce the time and cost required to reach higher quality care. This is particularly pertinent for women in rural locations of Malawi, who are more likely to have geographic or financial barriers to accessing larger, more well-equipped health facilities. Several studies in sub-Saharan Africa have shown that supply-side interventions to improve workforce training and facility infrastructure have reduced maternal mortality by increasing availability of emergency obstetric care.^34–36^ However, most quality improvement studies to date are small with short evaluation windows and limited capacity to assess sustainable impact on health outcomes.^37^ The major gaps in maternal care capacity in lower tier facilities in Malawi^8 9^ suggest substantial investments would be required to make high-quality delivery care available in all health facility tiers. Alternatively, demand-side interventions designed to connect women to centralised facilities may rely on improved availability of low-cost or free transportation, as the poorest women are often unable to meet these costs to reach quality care.^33^ Voucher schemes for transportation have been found to be cost-effective to increase facility deliveries and reduce maternal mortality,^38^ but evidence also shows that conditional cash transfer schemes may not equally reach the poor and least educated, and their impact on neonatal mortality may be limited by obstetric care quality.^39^ Some evidence suggests that maternity waiting homes may effectively bring higher risk women within reach of better quality care.^40^

However, demand-side interventions that focus on increasing access to high-quality care may not be definitive solutions if they do not address the complex underlying social determinants of access. The finding that poorer, less literate women do not demonstrate a preference for quality, as measured by facility capacity, points to a need to change the demand for care. Numerous studies have found an inverse relationship between patient education and patient satisfaction of nursing care,^40^ suggesting that increased education may create higher expectations for the patient experience. This has potentially powerful implications, given that word of mouth influences care-seeking behaviour.^41^

This study has some limitations. First, the MES asked women what type of facility they selected for delivery, but not the facility name. For this reason, women were assigned to the closest facility of the type that they chose based on the best available match; women who actually selected a more distant facility of the same type will be misclassified. The basic obstetric readiness score was used as a proxy for quality since it includes structural capacity measures, which are a component of quality, but it is a coarse measure, and its relationship to individual perceptions of quality and patient experience is unknown. Lastly, entropy reveals that separation between classes is limited. Facility type was excluded from the latent class analysis and multilevel model in further consideration of this measure and did reveal a higher entropy (0.19 and 0.67, respectively) or clearer separation between classes. However, author consensus deemed facility type to be central to the integrity of the model, given that facility type is the basis of the creation of each woman’s facility choice set. The model was not considered without the remaining facility-specific variables of distance, fees and obstetric readiness score as these are common determinants of facility selection based on existing literature. Additionally, it is noted that entropy is only one measure of model quality. The tests of model fit in support of our final model selection include the BIC, the percentage of the population represented in each class (no class has less than 1% of the population)^42^ and successful convergence.^42^ Study strengths include those of a large dataset that linked a nationally representative sample with detailed health system information using spatial location data and incorporation of the population’s heterogeneity into the latent class model.

Additional research is warranted to expand and contextualise these findings. Data that offer exact location of delivery for each woman may reveal additional preferences, or reinforce the validity of the assigned preference. Similarly, qualitative data from a sample of these women may provide insight into why these preferences exist. Data on what information women have about the quality of care provided at different facility types may also inform interpretations of their preferences. Lastly, this study highlights the need for routine collection of geospatial data on facilities in order to understand utilisation.^33^

Future policies designed to increase utilisation of high-quality obstetric care in Malawi will need to address distance and fees, as quality alone is unlikely to influence most women’s facility type selection, given the current revealed-preferences for care. This is especially critical given that women who are poor and illiterate are less likely to deliver in high-quality facilities, despite associations with reduced maternal and neonatal mortality. Potential interventions may target increasing quality of decentralised facilities, enhancing accessibility of high-quality centralised facilities through decreased user fees and improved transport, or altering patients’ demand for quality. Ultimately, to increase utilisation, women may need navigation around complex social barriers that deter them from seeking and reaching high-quality care.^43^
