# Supplementary material for: Latent class analysis of the social determinants of health-seeking behaviour for delivery among pregnant women in Malawi
Source: BMJ Glob Health. 2019 Mar 30;4(2):e000930. doi: 10.1136/bmjgh-2018-000930 (PMC6441245; doi:10.1136/bmjgh-2018-000930)
Supplement: Supplementary data [file bmjgh-2018-000930supp001.pdf]

Appendix, Table 1. Data dictionary

Survey question*	Variable name	Variable definition	Variable type	Categories
Household Information Panel: Cluster number MES HH1 (p. 541)	hh1	Enumeration area code/cluster number	Continuous	
Household Information Panel: Cluster number → household number → Woman's Information Panel: Woman's line number MES HH1: HH2: WM4 (p. 541 and 564)	w_id	Unique woman identification number	Continuous	
Calculated by survey administrators post-hoc	w_weight	Woman's sampling weight	Continuous	
Household Information Panel: Urban or Rural MES HH6 (p. 541)	urban	Urban residence	Binary	
Soap: When you have soap in the house, what do you use it for? Washing hands MES SD1 (p. 600)	hh_usesoaphw	Woman reports soap used for handwashing	Binary	
Calculated by survey administrators post-hoc	hh_windex5	Wealth quintile	Categorical	Poorest
				Poor
				Middle
				Rich
				Richest
Woman's background: In what month and year were you born?	w_agebirth	Woman's age (years) at birth of child	Continuous	

→ How old are you?<br>Birth History:<br>In what month and year was (*child's name*) born?<br>MES WB1, WB2, BH4<br>(p. 566, 569-70)				
Woman's background:<br>In what month and year were you born?<br>→ How old are you?<br>MES WB1, WB2<br>(p. 566)	w_age	Woman's age (years) today	Continuous	
Household Characteristics:<br>To what ethnic group does the head of this household belong?<br>MES HC1C (p. 551)	w_ethnicity	Ethnicity of head of household	Categorical	Chewa<br>Tumbuka<br>Lomwe<br>Tonga<br>Yao<br>Sena<br>Nkhonde<br>Ngoni<br>Other
Household Characteristics:<br>What is the religion of the head of this household?<br>MES HC1A (p. 551)	w_religion	Religion of head of household	Categorical	Catholic<br>CCAP<br>Anglican<br>Seventh Day Adventist<br>Other Christian<br>Muslim<br>No religion<br>Other religion
Woman's Background: Have you ever attended school?<br>What is the highest	w_elevel	Woman's education level	Categorical	None (includes preschool)<br>Primary<br>Secondary<br>Higher

level of school you attended?<br>MES WB3, WB4 (p. 566)				
Calculated from education level	w_edsec	Woman: secondary education or above	Binary	
Woman's Background: Now I would like you to read this sentence (show sentence on card to respondent) to me.<br>MES WB5, WB7 (p. 566)	w_literacy	Woman: result of literacy test	Categorical	Can read (Includes "Able to read whole sentence" and "Able to read only parts of sentence")
				Cannot read
				Blind/visually impaired
Marriage/Union:<br>Are you currently married or living together with a man as if married?<br>MES MA1 (p. 588)	w_mstatus	Woman's marital status	Categorical	Currently married (Includes "Yes, currently married", and "Yes, living with a man")
				Not currently married
Marriage/Union:<br>How old is your husband/partner?<br>MES MA2 (p. 588)	partner_age	Spouse or partner age (years)	Continuous	
Fertility/Birth History: Sum of number of live births<br>MES CM10 (p. 568)	w_ceb	Total children born	Continuous	
Maternal and Newborn Health:<br>Did you see anyone for antenatal care during your pregnancy with (*child's name*)?<br>MES MN1 (p. 573)	anc_any	Any antenatal care visit during this pregnancy	Binary	
Calculated from:	anc_4visit	≥ 4 antenatal care visits during this pregnancy	Binary	

Maternal and Newborn Health: How many times did you receive antenatal care during this pregnancy? MES MN3 (p. 573)				
Maternal and Newborn Health: For women who delivered by caesarean section: When was the decision made to have the caesarean section? Was it before or after your labour pains started? MES MN19, MN19A (p. 576)	del_cspredet	Cesarean delivery decided on before labor	Binary	
Calculated from 5 items in MES for woman's last pregnancy. Number of risk factors that each woman reported. Risk factors included maternal age <19, incomplete antenatal care (<4 visits), primiparity, giving birth to multiples (e.g., twins), and giving birth to a very small neonate (<2500g, or a neonate described as very	del_risk	Delivery risk score	Categorical	0 risk factors<br>1 risk factor<br>2 risk factors<br>3 risk factors<br>4 risk factors<br>5 risk factors

small if no birthweight data).)				
Birth History:<br>In what month and year was (*child's name*) born?<br>MES BH4 (p. 569-70)	inf_dob	Child date of birth	Continuous	
Calculated from infant date of birth, with rainy season from November through April	rainy	Child date of birth during rainy season	Binary	
Birth History:<br>Were any of these births twins?<br>MES BH2 (p. 569-70)	inf_multiple	More than one child birthed during delivery	Binary	
Birth History:<br>Line number<br>MES BH LN (p. 569-70)	inf_primip	Primiparous	Binary	
Desire for Last Birth:<br>When you got pregnant with (*child's name*), did you want to get pregnant at that time?<br>MES DB1 (p. 572)	inf_unintended	Woman's pregnancy was not wanted at all or at that time	Binary	
Desire for Last Birth:<br>If pregnancy was unintended: Did you want to have a baby later on, or did you not want any (more) children?<br>MES DB3 (p. 572)	inf_mistimed	Woman's pregnancy earlier than desired	Binary	
Desire for Last Birth:	inf_unwanted	Woman's pregnancy unwanted	Binary	

If pregnancy was unintended: Did you want to have a baby later on, or did you not want any (more) children?<br>MES DB2 (p. 572)				
Birth History:<br>Is (*child's name*) still alive?<br>MES BH5 (p. 569-70)	w_csurv	Number of surviving children out of total children ever birthed	Continuous	
Maternal and Newborn Health:<br>Where did you give birth to (*child's name*)? Choices for facility types were provided.<br>MES MN18 (p. 575)	MES_factype	MICS facility type where woman delivered	Categorical	Non-facility<br>Public<br>Private<br>Mission<br>Other
Facility Identification:<br>Type of facility (country-specific)<br>SPA 006<br>Appendix C (p. 186)	spa_factype	SPA facility type	Categorical	Central hospital<br>District hospital<br>Community hospital<br>Other hospital<br>Health center<br>Maternity<br>Dispensary<br>Clinic<br>Health post
Facility Identification:<br>Managing authority (ownership)<br>SPA 007<br>Appendix C (p. 186)	fac_mga	SPA facility management type	Categorical	Government<br>Private<br>CHAM
User Fees:<br>Does this facility have a fee for the following	fees_delivery	Delivery fees charged in facility	Binary	

items: Normal deliveries<br>SPA 362_07<br>Appendix C (p. 194)				
Composite calculated from 19 items on SPA facility audit	ssr_bobs_score	Service readiness: basic obstetric care	Continuous	
Calculated during analysis, see Methods	dist_km	Distance (kilometers) to facility	Continuous	

*Survey questions are taken from the Malawi MDG Endline Survey[18] or the Malawi Service Provision Assessment (SPA)[19], and exact survey question numbers and pages within those instruments are noted.

Appendix, Figure 1. Graph of Bayesian information criterion showing optimal number of classes for formula selection.

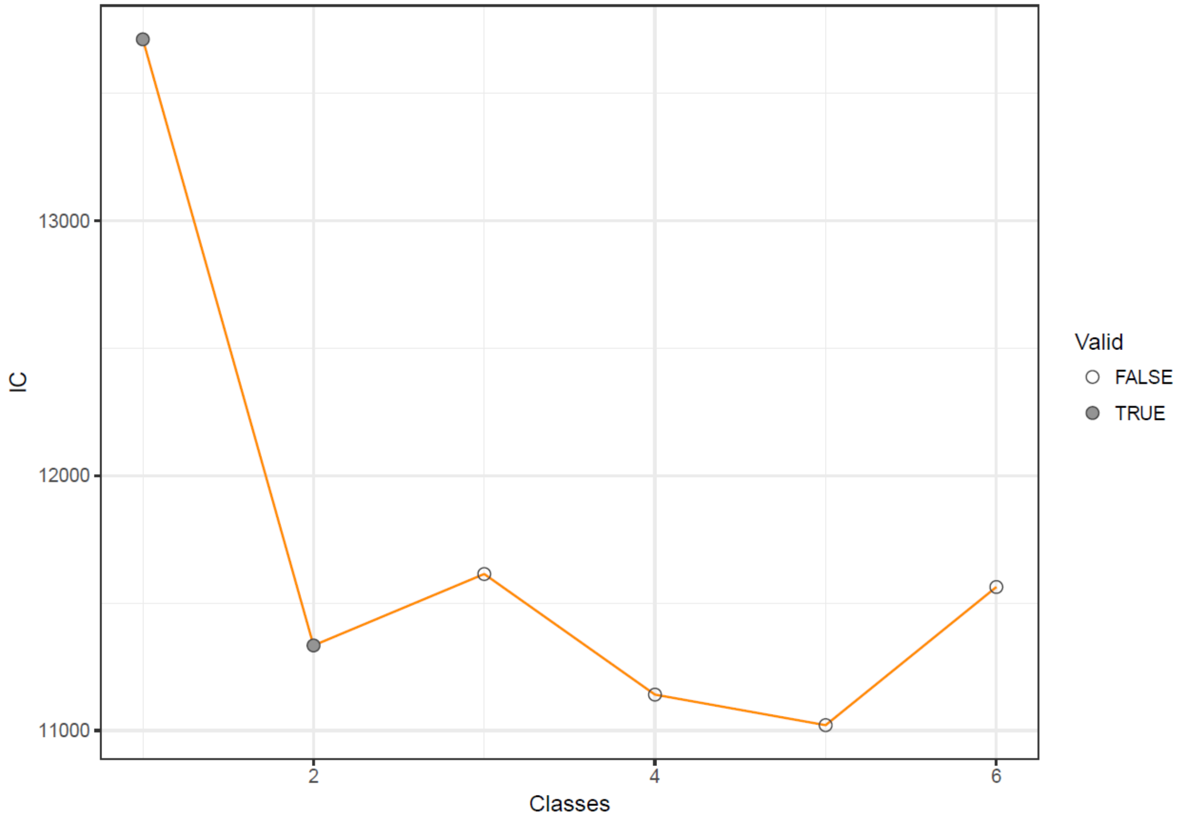

Validity was marked as FALSE when the formula was collinear, or computationally singular.

Appendix, Table 2. Comparison of coefficients to evaluate random effects

	Coefficient		
Alternative	Uncorrelated (Latent Class Model)	Correlated (Multilevel Model)	Uncorrelated (Latent Class) *p*-value
**Class 1 facility-level preferences**			
Facility type			
Central Hospital	Reference		
District hospital	14·492	-0·507	0·895
Community hospital	7·062	-321·908	0·949
Other hospital type	14·866	-87·238	0·893
Clinic	6·353	-249·587	0·954
Health center	15·045	-0·879	0·891
Maternity	13·779	-39·396	0·900
Distance to facility (km)	-6·050	-131·550	2·20E-16
Basic obstetric readiness (scale 0-1)	-0·434	-12·066	0·111
Fees (reference = no fees)	-5·735	-169·974	2·20E-16
Class 2 facility-level preferences			
Facility type			
Central Hospital	Reference		
District hospital	-1·054	6·592	7·82E-05
Community hospital	-2·242	-0·723	2·20E-16
Other hospital type	-2·393	-0·865	2·20E-16
Clinic	-2·862	-1·467	2·20E-16
Health center	-4·629	-3·324	2·20E-16
Maternity	-21·938	-150·845	0·996
Distance to facility (km)	0·834	0·181	2·20E-16
Basic obstetric readiness (scale 0-1)	1·441	3·173	9·14E-07
Fees (reference = no fees)	-0·105	-1·090	0·330
Individual-level characteristics of Class 2 compared to Class 1			
Wealth			
Poorest	Reference		
Poor	-0·072	-0·209	0·479
Middle	-0·004	-0·066	0·969
Rich	0·099	-0·150	0·362
Richest	0·316	0·403	0·018
Urban/Rural			
Rural	Reference		
Urban	-0·006	-0·792	0·963
Woman's age	0·140	0·125	0·036
Spouse or partner age	-0·091	-0·080	0·148
Education			

Primary education or below (includes preschool)	Reference		
Secondary education or above	0·487	0·519	3·50E-07
Literacy			
Literate	Reference		
Illiterate	-0·187	-0·224	0·018
Blind or visually impaired	12·659	58·854	0·945
Primiparous	0·294	0·342	0·095
Multiple birth (*e.g.*, twins)	1·010	1·562	4·74E-05
Woman's pregnancy unwanted	0·356	0·308	0·002
At least 4 antenatal care visits during pregnancy	0·000	0·060	0·998
Delivery risk score (scale 0-4)	-0·024	-0·058	0·830
Cesarean delivery planned before labor onset	2·431	3·658	3·29E-09
